# Identification of Potential Genes Encoding Protein Transporters in *Arabidopsis thaliana* Glucosinolate (GSL) Metabolism

**DOI:** 10.3390/life12030326

**Published:** 2022-02-22

**Authors:** Sarahani Harun, Nor Afiqah-Aleng, Fatin Izzati Abdul Hadi, Su Datt Lam, Zeti-Azura Mohamed-Hussein

**Affiliations:** 1 Centre for Bioinformatics Research, Institute of Systems Biology (INBIOSIS), Universiti Kebangsaan Malaysia, Bangi 43600, Selangor, Malaysia; zeti.hussein@ukm.edu.my; 2 Institute of Marine Biotechnology, Universiti Malaysia Terengganu, Kuala Nerus 21030, Terengganu, Malaysia; afiqahaleng@umt.edu.my; 3 Department of Biological Sciences and Biotechnology, Faculty of Science and Technology, Universiti Kebangsaan Malaysia, Bangi 43600, Selangor, Malaysia; p105328@siswa.ukm.edu.my; 4 Department of Applied Physics, Faculty of Science and Technology, Universiti Kebangsaan Malaysia, Bangi 43600, Selangor, Malaysia; sudatt@ukm.edu.my

**Keywords:** glucosinolate, co-expression network, molecular docking, transporter proteins

## Abstract

Several species in *Brassicaceae* produce glucosinolates (GSLs) to protect themselves against pests. As demonstrated in *A. thaliana*, the reallocation of defence compounds, of which GSLs are a major part, is highly dependent on transport processes and serves to protect high-value tissues such as reproductive tissues. This study aimed to identify potential GSL-transporter proteins (TPs) using a network-biology approach. The known *A. thaliana* GSL genes were retrieved from the literature and pathway databases and searched against several co-expression databases to generate a gene network consisting of 1267 nodes and 14,308 edges. In addition, 1151 co-expressed genes were annotated, integrated, and visualised using relevant bioinformatic tools. Based on three criteria, 21 potential GSL genes encoding TPs were selected. The AST68 and ABCG40 potential GSL TPs were chosen for further investigation because their subcellular localisation is similar to that of known GSL TPs (SULTR1;1 and SULTR1;2) and ABCG36, respectively. However, AST68 was selected for a molecular-docking analysis using AutoDOCK Vina and AutoDOCK 4.2 with the generated 3D model, showing that both domains were well superimposed on the homologs. Both molecular-docking tools calculated good binding-energy values between the sulphate ion and Ser419 and Val172, with the formation of hydrogen bonds and van der Waals interactions, respectively, suggesting that AST68 was one of the sulphate transporters involved in GSL biosynthesis. This finding illustrates the ability to use computational analysis on gene co-expression data to screen and characterise plant TPs on a large scale to comprehensively elucidate GSL metabolism in *A. thaliana*. Most importantly, newly identified potential GSL transporters can serve as molecular tools in improving the nutritional value of crops.

## 1. Introduction

Plants are sessile organisms that are regularly subjected to a variety of biotic and abiotic stresses, resulting in biochemical and physiological changes that have a significant impact on plant development and survival. In general, plants have two basic defence mechanisms to overcome these challenges: structural responses and metabolic changes [1,2]. The production of secondary metabolites is one of the metabolic changes that occur in response to both biotic and abiotic stresses [3]. Glucosinolates (GSLs) are an extensively studied group of secondary metabolites [4] due to their role as major defence compounds in plants, protecting against herbivores and pathogens [5]. GSLs are unique to the *Brassicaceae* family, and they are found in plants such as *Arabidopsis thaliana* and many cultivated vegetables (broccoli, cauliflower, cabbage, wasabi, horseradish, and mustard) [4,6,7]. The genotype, climate, and cultivation conditions, such as fertilisation and harvest time, all influence the composition and content of GSLs, and they are very diverse amongst the GSL containing plants [8]. GSLs are characterised by the existence of nitrogen and at least two sulphur atoms in the GSL core structure, suggesting that sulphur metabolism is essential in GSL biosynthesis [9]. 

GSLs are grouped based on their precursors (having different side chains). There are three GSL groups—aliphatic GSLs, which are produced from methionine, alanine, leucine, isoleucine, or valine; indolic GSLs, which are synthesised from tryptophan; and benzyl GSLs, which are produced from phenylalanine or tyrosine [10,11,12]—and more than 130 different GSLs in GSL-containing plants have been identified [13]. GSLs are water-soluble compounds that are stable when stored in plant cells [10]. Plant-cell disruption from insect feeding or mechanistic disruption leads to the GSL-myrosinase (GM) activation of nitriles, epithionitriles, isothiocyanates, and/or thiocyanates, which are converted from unstable aglycones, to protect plants against biotic and abiotic stresses [14,15]. This process is catalysed by myrosinases (TGG, EC 3.2.1.147), which also contribute to the variation of GSL products and GSL side-chain compositions [4,16]. The GM mechanism is known to release a toxic “mustard oil bomb” for repelling pathogens and insects [4,17]. The isothiocyanates, which appear to be universally toxic, have been attributed to the GM system’s defence role. When utilised in bioassays with insects in pure form, their toxicity is comparable to that of commercial insecticides [18].

GSLs are stored in several locations, such as the laticifer-like S-cells and along the leaf margin and seeds [19,20,21,22,23]. The composition of GSLs differs quantitatively and qualitatively in different GSL-containing plant organs. The GSL concentrations are generally higher in roots than shoots [24]. Previous studies suggested that the distribution of GSLs across various plant organs can be explained by the optimal defence theory [25]. Compounds involved in a defence mechanism are preferentially distributed to organs that are more attractive to pests [26,27]. Thus, the reproductive organs, such as the flowers and seeds, store the highest concentrations of GSL, while the concentrations of GSL in the tissues below ground are the highest in the tap and lateral roots [28]. 

In GSL metabolism, sulphated GSLs are produced in the cytoplasm and stored in the vacuoles or S-cells in the periphery of the phloem [29]. The GSL concentration in S-cells is up to 20 times higher than that in the surrounding tissues [30]. However, the transport mechanism for GSL storage in both vacuoles and S-cells is still unclear [31]. A proteomic analysis on S-cell cytoplasm conducted by Koroleva et al. [20] failed to identify the existence of GSL-biosynthetic enzymes, hence suggesting the involvement of transporters in the accumulation of GSLs in the cells [20]. Transporter proteins (TPs) play an essential role in various mechanistic properties in plants, such as signalling, metabolism, and physiology involving the translocation of different molecules (hormones, amino acids, sugars, inorganic ions, water, and solutes) through plant membranes [32]. At present, five TPs have been experimentally validated as being involved in various GSL metabolisms, i.e., GSL transporters (GTR1 and GTR2), sulphate transporters (SULTR1;1 and SULTR1;2), and sodium symporter family protein 5 (BAT5) [12].

The long-distance transportation of a GSL from its source (leaf, root, and silique) to where it accumulates (leaf, root, silique, and embryo) is regulated by GSL TPs (GTRs) [33,34,35]. Nour-Eldin et al. [33] found that the *Arabidopsis* nitrate/peptide group GSL transporters AtNPF2.10 (AtGTR1) and AtNPF2.11 (AtGTR2) were responsible for the long-distance transport of short- and long-chain aliphatic GSLs from source tissues to target tissues. GTR1 and GTR2 facilitate long-distance GSL transport through phloem and xylem tissues [33,34,36,37]. Andersen et al. [34] conducted a micro-grafting experiment in *Arabidopsis* and found a specific GSL transporter that facilitates the transport of indole GSLs between the rosette leaves and roots [37]. Meanwhile, Madsen et al. [38] found that leaves of *Arabidopsis gtr1gtr2* mutants reduced the fitness of green peach aphids (*Myzus persicae*) by reducing the availability of GSL in phloem sap and increasing GSL in the tissues around the phloem. This observation suggests a potential application for these transporters in novel resistance against the insect. Detailed understanding of the defence mechanism involving GSLs can facilitate the development of crops that are more resistant to pests [35,38].

The sulphur uptake in GSL-containing plants suggests a significant role for sulphur in GSL biosynthesis. Metabolomic and transcriptomic studies by Koprivova and Kopriva [39] and Morikawa-Ichinose et al. [40] found that GSL accumulation was significantly reduced in a sulphur-deficient environment, suggesting a role for sulphur in GSL biosynthesis [41,42,43]. SULTR1;1 and SULTR1;2 are sulphate transporters found in *Arabidopsis* roots, and their expression is increased during sulphur limitation [44]. The sulphate transportation and GSL-transport machinery mechanism is more complex in *Brassica* crops. Thus, elucidating this mechanism is essential, as its knowledge can be used to design transporters as molecular tools in crop improvement. Another known GSL TP, BAT5, functions specifically in the side-chain elongation of aliphatic GSL biosynthesis by directing 2-oxo acids into the chloroplast. The involvement of the chloroplastic TP in the GSL mechanism was shown by depleting the function of *BAT5* in a *bat5*-knockout *Arabidopsis* mutant, in which the level of aliphatic GSL was reduced [45,46]. Thus, GSL biosynthesis is known to occur within plastids and the cytoplasm.

Over the last few decades, genome sequencing, combined with rapid advancement in bioinformatics has led to comprehensive molecular studies on the model plants as well as non-model plants with economic importance. Furthermore, the application of bioinformatics can be used to elucidate the complex biological processes generated from molecular datasets that would unravel hypotheses of the gene’s functions, protein interactions, and other molecular mechanisms efficiently [47,48,49]. However, public databases host inaccurate information on the putative roles of TPs due to various limitations in the gene- and protein-sequence annotation processes and erroneous mismatching between genomic and functional data on protein function. Therefore, we propose a ‘guilt-by-association’ (GBA) approach to identify and characterise possible GSL TPs involved in GSL metabolism [32]. The GBA principle has been used to identify regulators [50,51,52,53,54,55] and enzymes [56,57,58,59] involved in GSL biosynthesis. Detailed information on most of the molecular components related to GSL biosynthesis and metabolism can be found in SuCComBase, which is accessible at http://plant-scc.org (accessed on 16 December 2021) [60].

In this paper, we describe the process for searching for potential GSL genes that may be involved in the transportation of GSL-related components in *A. thaliana*. Firstly, a GSL co-expression network was constructed in search of the co-expressed genes, which was followed by GO enrichment analysis to infer the function of the identified potential GSL genes. Three criteria were designed to facilitate the selection of potential GSL TPs: (i) involvement in transport and localisation, (ii) sharing similar expression patterns with known GSL genes, and (iii) having similar subcellular localisation with known GSL TPs. 

## 2. Results

### 2.1. Data Collection and Establishment

**Figure 1:**
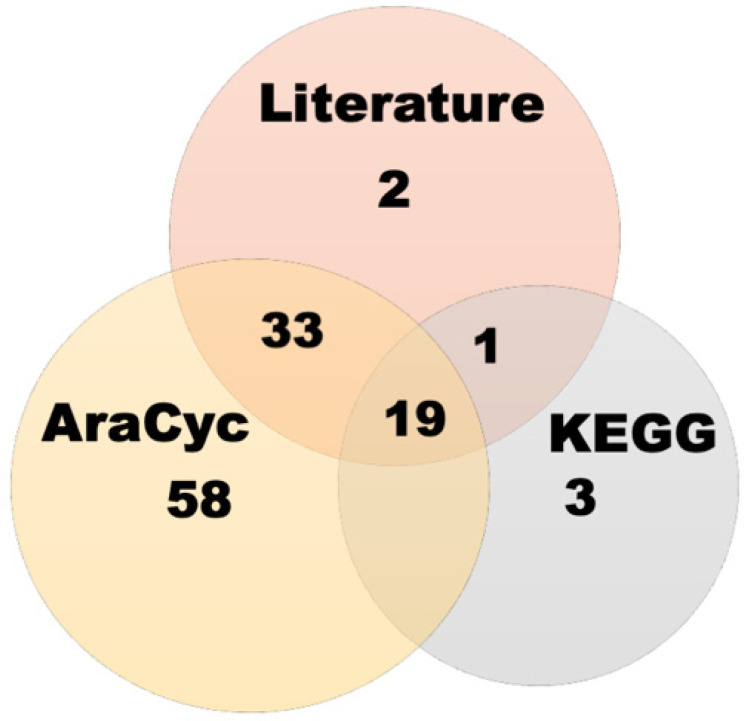
Identification of GSL genes from the literature, KEGG, and AraCyc.

A total of 188 known GSL genes were identified from the literature (55 genes), KEGG (23 genes), and AraCyc (110 genes); however, only 116 were used in this analysis after redundancies were removed. The GSL genes were identified using “glucosinolate” and “GSL” as keywords in the search tab of each database and specifically selecting *Arabidopsis thaliana* datasets (Figure 1). The complete list of known GSL genes is shown in Supplementary Table S1. 

### 2.2. Gene Co-Expression Network Analysis

**Figure 2:**
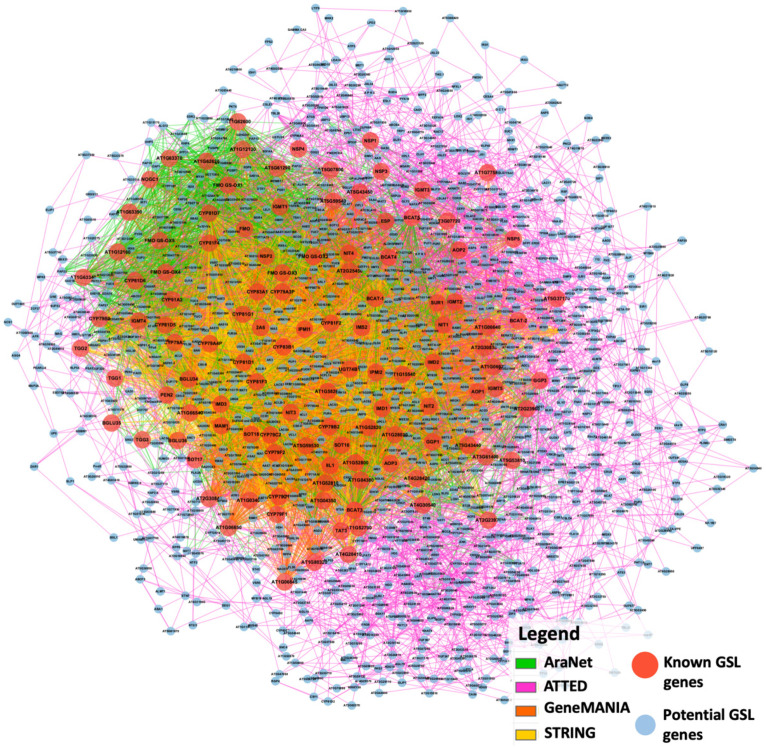
Integrated co-expression network of GSL genes consisting of 1267 nodes and 14,308 edges.

All 116 known GSL genes were used as queries against transcriptomic data from the four specified co-expression network tools, including ATTED-II (http://atted.jp/ (accessed on 16 February 2021)) [61], AraNet v2 (https://www.inetbio.org/aranet/ (accessed on 16 February 2021)) [62], GeneMANIA (https://genemania.org/ (accessed on 16 February 2021)) [63,64], and STRING (https://string-db.org/ (accessed on 16 February 2021)) [65,66,67]. All the identified interactions were combined to form a single gene co-expression network using Cytoscape 3.8.2 [68]. Figure 2 shows the interaction between 116 known GSL genes with 1151 potential GSL genes linked with 14,308 edges. The potential GSL genes are defined as the identified co-expressed genes in the gene network. The integrated co-expression network was generated from 293 nodes and 2265 edges from AraNet; 932 nodes and 2894 edges from ATTED; 213 nodes and 9910 edges from GeneMANIA; and 211 nodes and 4470 edges from STRING. These networks of the individual genes were merged using Cytoscape 3.8.2, resulting in an integrated co-expression network consisting of 1267 nodes and 14,308 edges linking the genes (Figure 2).

### 2.3. GO Enrichment

**Figure 3:**
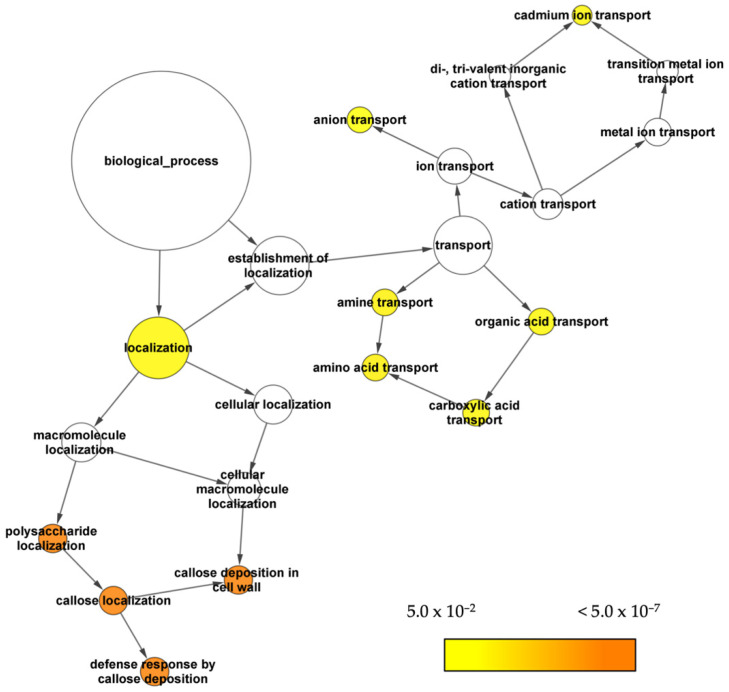
The overrepresented localisation biological processes of GO in the co-expressed GSL genes from the gene network. The significance levels of the overrepresented GO terms are shown using a heatmap, where darker nodes mean more significant ontologies.

BINGO was used to analyse the GO enrichment on the constructed gene network. We used the overrepresented GO biological processes (Figure 3) as a guide to search for potential genes that encoded transporter proteins involved in GSL metabolism. The GO enrichment analysis showed that the nodes on localisation and transport were among the overrepresented biological processes. 

### 2.4. Gene-Expression Pattern Analysis and Visualisation

**Figure 4:**
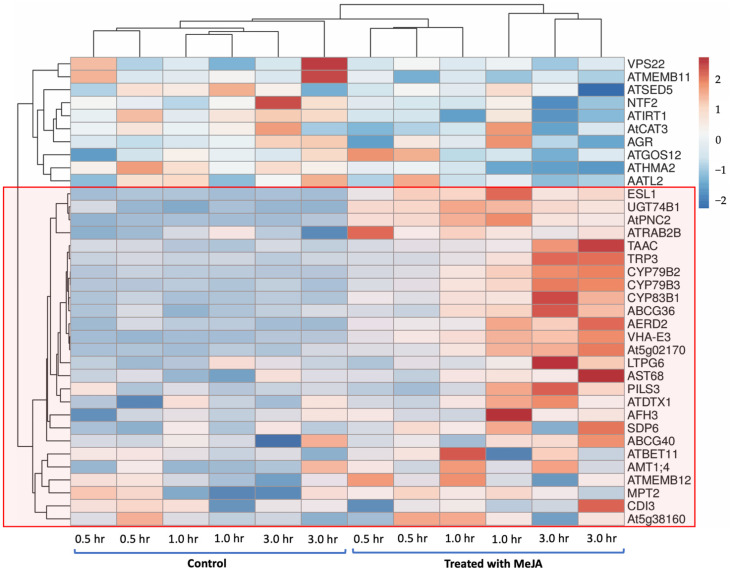
The expression patterns for known GSL genes (*UGT74B1*, *CYP79B2*, *CYP79B3*, *CYP83B1*, and *ABCG36*) and potential GSL genes encoding TPs in control and MeJA–treated conditions in *A. thaliana*.

We used jasmonic-acid-treated *A. thaliana* gene-expression data obtained from Expression Angler (http://bar.utoronto.ca/ExpressionAngler/ (accessed on 12 April 2021)) to validate the potential genes identified in this study. Gene-expression data for *A. thaliana* wild type Col-0 were collected at 30 min, 1 h, and 3 h time points in both control and MeJA-treated conditions. The expression patterns for the selected potential GSL genes compared to those for known GSL genes were generated using ClustVis (https://biit.cs.ut.ee/clustvis/ (accessed on 12 April 2021)) [69] (Figure 4), and known GSL genes were grouped based on their function in GSL biosynthesis. The expression patterns in Expression Angler were calculated using *r*-values based on Pearson’s correlation coefficient (PCC). Figure 4 shows that the expression patterns for known GSL genes (*UGT74B1*, *CYP79B2*, *CYP79B3*, *CYP83B1*, and *ABCG36*) were similar to those for 21 potential GSL genes encoding TPs in control and treated conditions (MeJA) in *A. thaliana*.

### 2.5. Sequence Analysis of Potential GSL TPs

The subcellular location information of each potential GSL TP was retrieved from the SUBA4 database. The information extracted for the known GSL TPs was used as a reference for protein structural analysis to predict the function of those potential GSL TPs in the GSL-biosynthesis pathway. Supplementary Table S2 shows the results collected from various databases such as TAIR, UniProt, GO, and SUBA4. Twenty-one potential GSL genes encoding TPs have expression patterns similar to those of known GSL genes and are associated with localisation and transport processes, including *ESL1*, *AtPNC2*, *AtRAB2B*, *TAAC*, *AERD2*, *VHA-E3*, *At5g02170*, *AST68*, *PILS3*, *AtDTX1*, *ABCG40*, *AtBET11*, *AMT1;4*, *AtMEMB*, *MPT2*, *CDI3*, *TRP3*, *LTPG6*, *At5g38160*, *AFH3*, and *SDP6.* Then, *AST68* and *ABCG40* were selected for further analysis, as they have subcellular localisations similar to those of known GSL TPs, i.e., *SULTR1;1* and *SULTR1;2*, and *ABCG36*, respectively.

### 2.6. Evolutionary Relationship Analysis of the Potential GSL TPs

**Figure 5:**
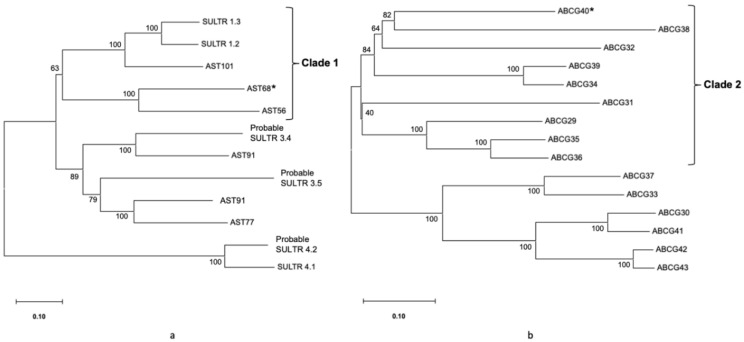
The phylogenetic trees of the selected potential GSL TPs indicated with the symbol “*”. (**a**) Phylogenetic tree of AST68 and its related sequences. (**b**) Phylogenetic tree of ABCG40 and its homologs.

Two phylogenetic trees of selected potential GSL TPs (AST68 and ABCG40) and their related sequences were constructed using MEGA11 (Figure 5). AST68 was located in the same clade (clade 1) with known GSL TPs, i.e., SULTR1;1 (AST101) and SULTR1;2. Meanwhile, ABCG40 was grouped into known GSL TPs, i.e., ABCG36 or PEN3 in clade 2.

### 2.7. Protein Structure Prediction and Model Evaluation

AST68 and ABCG40 contain 677 and 1423 amino acids, respectively. Possible homologous structures for AST68 are solute carrier family-12 member (PDB ID 7CH1_B), solute carrier family-26 member (PDB ID 6RTC_A), and sulphate transporter (PDB ID 5DA0_A), whilst the ATP-binding cassette sub-family G members (PDB IDs 5DO7_D, 5DO7_C, and 6HZM_A) are homologs of ABCG40. The sequence identity of those homologs is within 20–30%; hence, threading and ab initio approaches were used to predict the tertiary structure of GSL TPs. However, the length of ABCG40 exceeded the maximum number of 1000 amino acids required by most servers; therefore, only the sequence numbers 506–1415 that contain transmembrane and cytoplasmic domains were retained.

**Figure 6:**
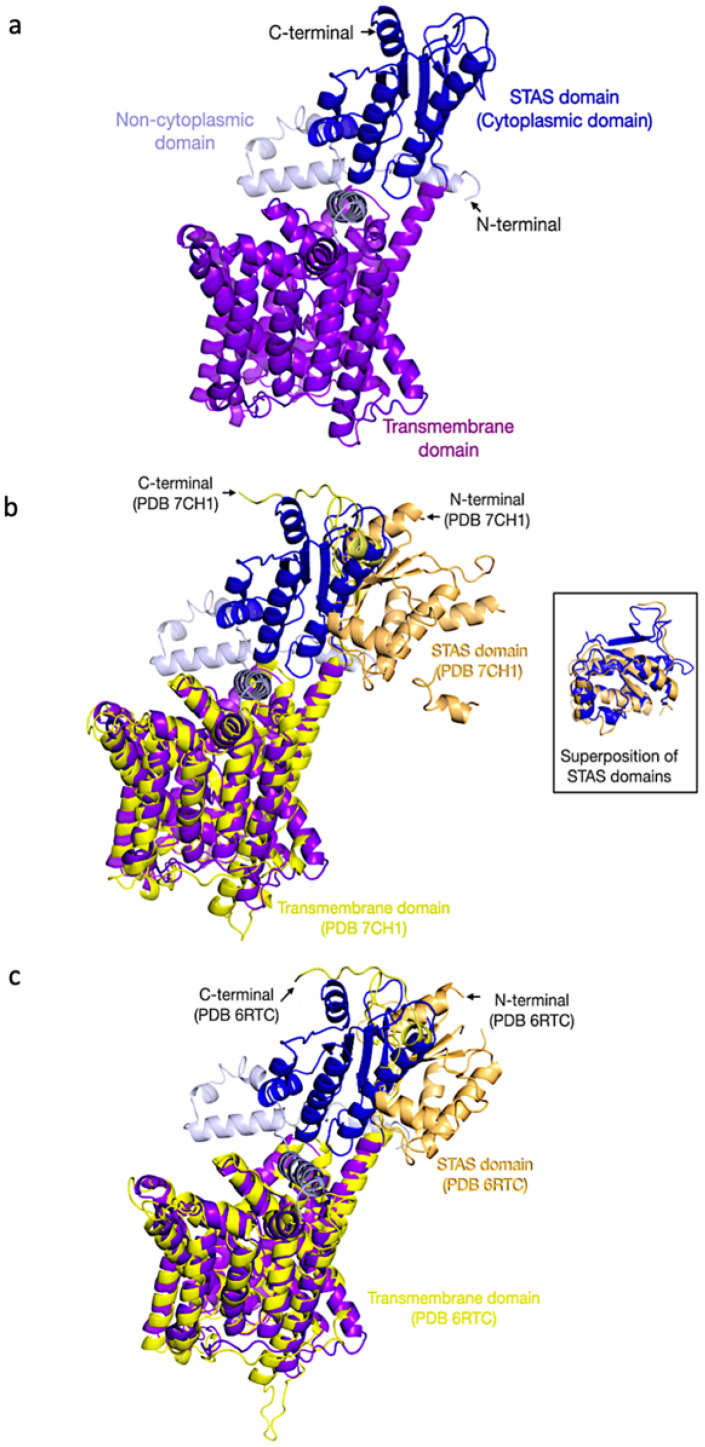
The 3D model of AST68 trRosetta and its homologs. (**a**) 3D structure of AST68 model. (**b**) Superimposition of AST68 3D model with SLC29A9 (PDB ID 7CH1), and superimposition of STAS domain is shown in the box. (**c**) Superimposition of AST68 model with SLC26A9 (PDB ID 6RTC).

trRosetta generated the best 3D models for AST68 and ABCG40, as shown from the MolProbity score, Ramachandran plot, and clashscore (Table 1). A comparison between the 3D model of AST68 (Figure 6a) and its homologs (i.e., SLC29A9 (PDB: 7CH1) and SLC26A9 (PDB: 6RTC)) showed that both domains (i.e., the STAS domain and transmembrane domain) were well superimposed on their homologs (7CH1 (Figure 6b) and 6RTC (Figure 6c)) even though the orientation of the whole structure was different, as shown by the RMSD values (3.65—PDB 7CH1; 3.69—PDB 6RTC). However, a comparison of the 3D model of ABCG40 against its homologs (PDB: 5NJ3, 6HCO, and 5DO7) showed that one of the domains did not superimpose well on its homolog. Thus, the generated 3D model of ABCG40 was eliminated from the molecular-docking analysis. 

### 2.8. Molecular Docking of AST68 with Sulphate Ion

**Figure 7:**
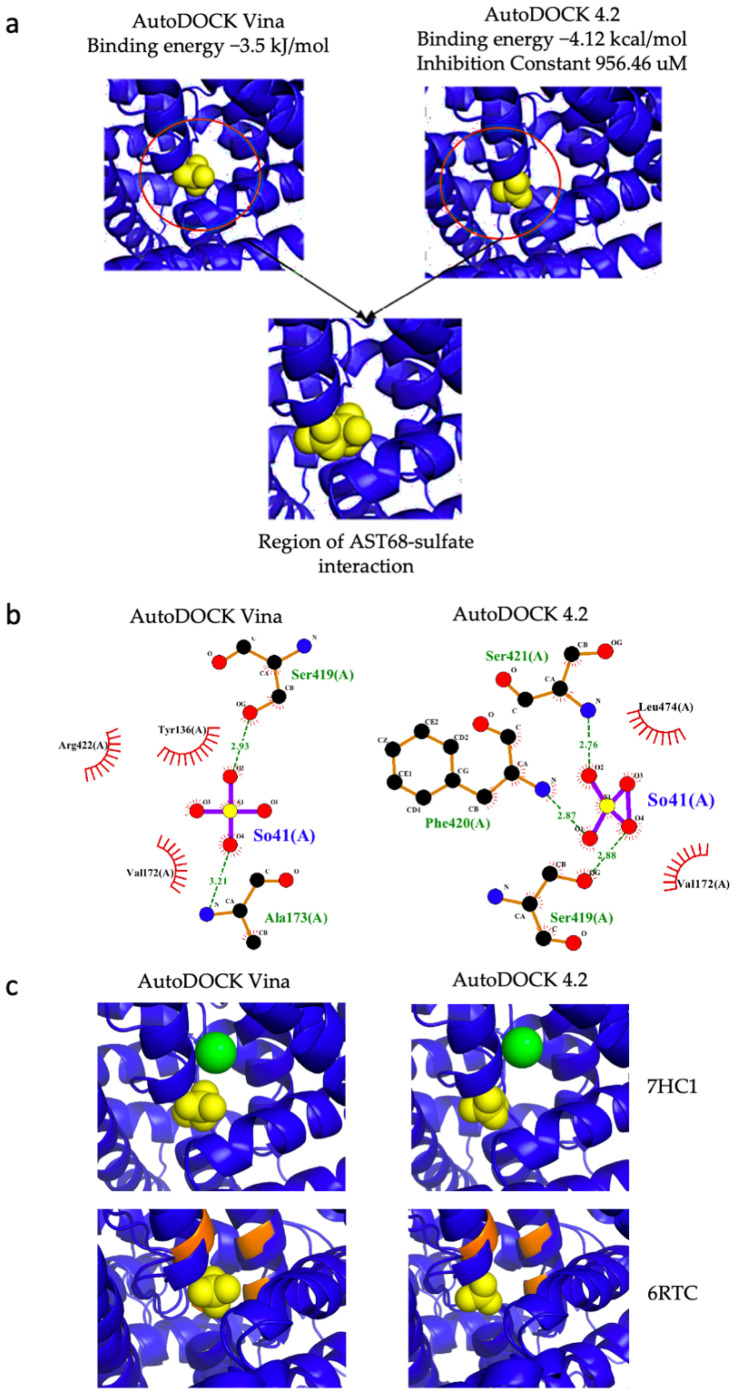
AST68–sulphate interaction. (**a**) Docking of sulphate ion on AST68 ion using AutoDOCK Vina and AutoDOCK 4.2 predicted similar interaction region. (**b**) LigPlot showing hydrogen-bonding interactions of AST68 and sulphate ion with Ser419 and Val172 as predicted by both approaches. (**c**) Comparison with AST68 and its homologs (7CH1 and 6RTC). In 7CH1, the sodium ion is coloured green, and the chloride-binding region in 6RTC is coloured orange.

The sulphate ion was docked onto AST68 using AutoDOCK Vina and AutoDOCK 4.2 and showed good binding-energy values of −3.5 kJ/mol and −4.12 kcal/mol, respectively (Figure 7a). Both tools predicted the interactions of the sulphate ion with Ser419 (forming a hydrogen bond) and Val172 (forming a van der Waals interaction) of AST68 (Figure 7b). The sulphate ion bound to the AST68 homolog at the region close to the sodium-ion (in 7CH1)- and chloride-ion-binding regions (in 6RTC) (Figure 7c).

## 3. Discussion

There are several limitations in characterising potential GSL TPs responsible for GSL metabolism. First, there remains some inaccurate information in biological databases regarding the roles of TPs. Thus, the annotation of genes and proteins with putative roles in TPs appears to face erroneous matching between genomic and functional data on protein function. Second, this limitation also affects the capability of using traditional homology-based approaches to categorise the TP features and assign the TP substrate specificity information to the physiological details of plants [32]. Therefore, several criteria have been used to identify and select potential GSL TPs: (1) potential TPs that are involved in transport and localisation from the GO analysis; (2) potential GSL genes encoding TPs that share similar expression patterns with known GSL genes in control and treated conditions (MeJA) in *A. thaliana*; and (3) potential TPs that have subcellular localisation similar to that of known GSL TPs. These criteria have been described by Larsen et al. [70]. It also highlighted the in silico-based approaches that employed the ‘guilt-by-association’ (GBA) principle in identifying transporters in plant specialised metabolism. In relation to GSL biosynthesis, the GBA approach has been used to identify regulators [50,51,52,53,54,55] and enzymes [56,57,58,59]. Identifying TPs using co-expressed genes successfully defined a boron transporter candidate in *A. thaliana* [71]. A similar approach was employed in the non-model plant *Catharanthus roseus*, wherein *CrNPF2.9* was co-expressed in the mono-indole alkaloid (MIA) pathway [72]. To our best knowledge, this is the first study reporting the application of co-expressed genes to identify potential TPs in GSL metabolism. The abundance of publicly available *Arabidopsis* microarray and RNAseq data facilitates the development of in silico techniques to identify candidate genes based on their co-expression with other known genes involved in similar biological processes of interest [70]. Supplementary Figure S1 shows a complete step-by-step procedure for identifying potential genes encoding TPs involved in GSL metabolism.

Twenty-one potential TPs related to transport and localisation have been retrieved, and gene-expression pattern analysis was conducted. We used an expression-based approach to search for genes that encode the GSL transporter in the GSL mechanism. An expression-based approach is usually used to identify transporters from differential-expression patterns in the specialised metabolism of the plant under various conditions or stresses [73]. Bioinformatic analyses were conducted on the genes before and after treatment with methyl jasmonate (MeJA) to observe their expression or response profiles relative to those for known GSLs. JA stimulation causes a mechanism response (movement, secretion, the production of enzymes, and gene expression). In addition, the JA exposure of plants can stimulate secondary-metabolite production. These metabolites play an essential role in plants’ responses and adaptation to their natural environment [73]. Based on Figure 4, 21 potential GSL genes encoding TPs shared similar expression patterns with the known GSL genes (*UGT74B1*, *CYP79B2*, *CYP79B3*, *CYP83B1*, and *ABCG36*) shown in the red box. For additional protein characterisation, the subcellular location of each putative GSL TP was collected from the SUBA4, TAIR, UniProt, and GO databases.

Next, we selected two potential GSL TPs with the possible substrates in the GSL-biosynthesis mechanism that fulfilled the three criteria in this study: (1) AST68 and (2) ABCG40 (Table 2). The two genes are involved in both transport and localisation, based on GO analysis. They share a similar expression pattern with known GSL genes in control and MeJA-treated conditions in *A. thaliana.* They also have similar subcellular localisation to known GSL TPs. AST68, known as sulphate transporter 2;1, is located in the plasma membrane, similar to SULTR1;1 and SULTR1;2. These two TPs are involved in the GSL sulphur-assimilation process that transports sulphate to the *Arabidopsis* roots [39,40]. The gene expression of *SULTR1;1* and *SULTR1;2* is significantly increased in *Arabidopsis sdi1sdi*-knockout lines. The sulphur-deficiency-induced genes *SD1* and *SD2* are major repressors that control GSL biosynthesis during sulphur deficiency [43]. In the phylogenetic tree, the potential GSL TP was positioned in the same clade (clade 1) as SULTR1;1 (AST101) and SULTR1;2 (Figure 5a), suggesting its possible involvement in GSL sulphur assimilation. 

Another potential GSL TP is the ABC transporter G family member 40 (ABCG40), which is located in the plasma membrane. ABCG40 belongs to the same subfamily as ABCG36. ABC transporters are located in most membranes (e.g., the plasma membrane) and found in all living organisms [74]. There are several types of substrates for this transporter group: small molecules (heavy metals, inorganic acids, and peptides), large molecules (lipids, polysaccharides, and steroids), and intact proteins [75,76]. In plants, these transporters are involved in diverse biological processes, such as responses to pathogens, diffusion-barrier formation, and phytohormone transport [76]. Meanwhile, ABCG36 or PEN3 was proposed to transport distinct indole-derived metabolites once the plant was attacked by pests in the indolic GSL-biosynthetic pathway. In the study, 4-O-β-d-glucosyl-indol-3-yl formamide (4OGlcI3F) was found to be abundant in *pen3 Arabidopsis* leaf, known as pathogen-inducible compounds. Thus, the PEN3 substrate was suggested to be the precursor of 4OGlcI3F for resistance against pests in *Arabidopsis* [77]. However, the underlying mechanism in transporting small molecules across the plasma membrane remains unknown [78]. Figure 5b shows the location of ABCG40 in clade 2, relative to its homologs. A known GSL TP, ABCG36, was found in the same clade as ABCG40, suggesting its possible role as a GSL TP in indolic GSL metabolism. 

The 3D protein models of both potential GSL TPs were constructed using trRosetta. The structural analysis of the models against their homologs suggested further analysis of the AST68 model due to the well-superimposed domains of STAS and the transmembrane on the known structures, i.e., 7CH1 (Figure 6b) and 6RTC (Figure 6c). In addition, results from the ModFOLD8 analysis (significant confidence e-value of 1.255 × 10^−4^) also suggested the suitability of the docking of sulphate ions on AST68. Furthermore, Chi et al. [79] and Walter et al. [80] demonstrated the ligand’s tendency to bind to the transmembrane domain. Thus, we docked the sulphate ion onto the transmembrane domain. Both molecular-docking tools (AutoDOCK Vina and AutoDOCK 4.2) calculated good values of binding energy between the sulphate ion and Ser419 and Val172, with the formation of a hydrogen bond and van der Waals interaction, respectively. 

Our proposed in silico-based approaches facilitated the discovery of several potential GSL TPs, which can be experimentally validated. However, due to the limited capability of identifying possible substrates for potential GSL TPs, we selected proteins with similar protein families to the known GSL TPs, including the sulphate transporters (SULTR1;1 and SULTR1;2) and the ABC transporter G family member 36 (ABCG36) or PENETRATION 3 (PEN3). These potential GSL TPs should be validated further using targeted mutation techniques conducted on the model plant, *A. thaliana*. As a result, this knowledge can be applied in other GSL-containing plants producing better yields and showing greater stress tolerance against pests for crop improvement. 

## 4. Materials and Methods

### 4.1. Data Collection and Construction of the Gene-Co-Expression Network

A comprehensive literature search was performed using relevant literature databases, including PubMed, Google Scholar, and Science Direct. Several relevant keywords (e.g., “glucosinolate” and “glucosinolate pathway”) were queried to find known GSL genes. Pathway databases, including Kyoto Encyclopedia of Genes and Genomes (KEGG) (http://www.genome.jp/kegg/ (accessed on 11 February 2021)) [81,82] and AraCyc (https://www.arabidopsis.org/biocyc/(accessed on 11 February 2021)) [83], were used in querying those databases for the known GSL genes used in this study, searching with the keywords search “glucosinolate” and “GSL”. These known GSL genes were used as queries for four co-expression tools—ATTED [61], AraNet v2 [62], GeneMANIA [64,65], and STRING [61]—to identify “additional” co-expressed genes. ATTED is a dedicated co-expression database exclusively for plants for unravelling functionally related genes [61]. AraNet v2, GeneMANIA, and STRING interactions are based on integration from experiments and computational predictions that include co-expression data [62,63,66]. “Additional” genes are defined as potential GSL genes based on the ‘guilt-by-association’ principle. An integrated gene network was constructed using Cytoscape 3.8.2 [68].

### 4.2. GO Enrichment Analysis

Gene ontology (GO) analysis was conducted using Cytoscape 3.8.2 with the Biological Network Gene Ontology (BiNGO) plugin [84] to determine the overrepresented GO categories. In addition, a hypergeometric test with a Benjamini and Hochberg false-discovery rate (FDR) was performed using the default parameters for adjusted *p*-values [85].

### 4.3. Expression-Pattern Analysis

Expression Angler (http://bar.utoronto.ca/ExpressionAngler/ (accessed on 12 April 2021)) was used to obtain relevant information on the genes of interest with similar expression or response profiles in specific conditions or treatments [86]. The expression profiles were extracted from Expression Angler, and the heatmap was generated using ClustVis (https://biit.cs.ut.ee/clustvis/ (accessed on 12 April 2021)) [69].

### 4.4. Characterisation of Potential GSL TPs

Protein sequences were retrieved from the TAIR10 (The Arabidopsis Information Resource) and UniProt [87] databases for protein-sequence analysis and characterisation. In addition, the SUBA database (The Subcellular Localization of Proteins in Arabidopsis Database) was used to predict the cellular localisation of TPs [88]. Different locations of TPs are presumed to carry different types of GSL derivatives. For example, one known GSL TP, BAT5, is found in chloroplasts and facilitates the localisation of 2-oxo acids from cytosol chloroplasts [45,46]. 

### 4.5. Sequence Analysis of GSL TPs

The following analysis was conducted for (1) potential TPs associated with transport and localisation from the GO analysis, (2) potential GSL genes encoding TPs that had expression patterns similar to those of known GSL genes in control and treated conditions with methyl jasmonate (MeJA) in *A. thaliana*, and (3) potential TPs that had subcellular localisation similar to that for known GSL TPs. 

### 4.6. Construction of Phylogenetic Tree

The protein sequences of the selected GSL TPs were used as queries for a sequence-similarity search using BLASTP at https://blast.ncbi.nlm.nih.gov/Blast.cgi (accessed on 30 December 2021) [89]. The UniProt database and *Arabidopsis thaliana* were selected against the annotated protein sets and between the *A. thaliana* paralogs as queries using default parameters. The retrieved sequences were subject to multiple sequence alignments using MAFFT at https://www.ebi.ac.uk/Tools/msa/mafft/ (accessed on 31 December 2021) [90]. The aligned sequences were used to construct phylogenetic trees using the neighbour-joining method in the MEGA software (version 11) [91,92]. One thousand replicates were used to obtain bootstrapping values in the constructed phylogenetic trees.

### 4.7. Protein-Structure Prediction and Model Evaluations

The tertiary-structure prediction of GSL TPs was conducted using threading and ab initio methods. The models were predicted by I-TASSER [93], Robetta (trRosetta) [94], and Raptor-X [95]. The quality check for each model was evaluated using the MolProbity score [96], Ramachandran plot, and Clashscore. ModFOLD8 [97] was used to calculate the best scoring model.

### 4.8. Molecular Docking of Potential GSL Transporters

The structure and relevant information of the potential substrate for GSL TP were obtained from the PubChem database [98]. Molecular docking between GSL TP and its substrate was performed using AutoDOCK Vina [99] and AutoDOCK 4.2 [100] to obtain a consensus prediction of the binding-site region.

## 5. Conclusions

This study demonstrated the use of a computational approach to identify potential GSL TPs from co-expression data. The selected genes coding for TPs (*AST68* and *ABCG40*) were identified using three criteria that were used in the selection process: (a) involvement in transport and localisation biological processes, (b) sharing similar expression patterns with known GSL genes, and (c) having subcellular localisation similar to that of known GSL TPs. The application of these criteria was based on the ‘guilt-by-association’ (GBA) principle to identify and characterise possible GSL TPs efficiently. Two 3D models were generated, and further analysis was conducted on AST68 due to the well-superimposed essential domains of the homologs. The molecular-docking study was conducted on the 3D model of AST68 to determine its interaction with the sulphate ion to support its function as a sulphate transporter in GSL metabolism. The results from this study could be experimentally validated in the targeted verification of gene expression and metabolite data in *A. thaliana*. Furthermore, applying this bioinformatics approach will increase the ability to screen and characterise plant TPs on a large-scale basis to understand the mechanical properties of GSL metabolism in *A. thaliana*.

## Figures and Tables

**Table 1:** Scores of the potential GSL TP structures predicted by three different servers.

Potential GSL TP	Server	MolProbity Score	Percentage of Residues That Fall Inside Ramachandran-Favoured Regions (%)	Clashscore, All Atoms
AST68	I-TASSER	3.29	80.15	15.6
	Robetta server (trRosetta)	1.38	96.89	4.13
	Raptor-X	5.00	94.67	488.12
ABCG40 (506–1415)	I-TASSER	3.60	65.94	18.74
	Robetta server (trRosetta)	1.37	97.49	5.04
	Raptor-X	4.93	95.31	448.78

**Table 2:** Selected potential GSL TPs with possible substrates in the GSL-biosynthesis mechanism.

Known GSL TP (TAIR ID/UniProt ID)	Potential GSL TP (TAIR ID/UniProt ID)	Localisation	Possible Substrate
SULTR1;1 (At4g08620/Q9SAY1) and SULTR1;2 (At1g78000/Q9MAX3)	AST68 (At5g10180/O04722)	Plasma membrane; sulphate transporter 2;1	Sulphate
ABCG36/PEN3 (At1g59870/Q9XIE2)	ABCG40 (At1g15520/Q9M9E1)	Plasma membrane; ABC transporter G family member 40	4OH-X

## Data Availability

Not applicable.

## Supplementary Materials

The following supporting information can be downloaded at: https://www.mdpi.com/article/10.3390/life12030326/s1. Figure S1: The step-by-step procedure to identify potential genes encoding GSL TPs; Table S1: List of known GSL genes identified from the literature, KEGG, and AraCyc; Table S2: List of potential GSL transporters based on gene-expression patterns with known GSL genes and biological processes.
