# Supplementary material for: Identification of Potential Genes Encoding Protein Transporters in Arabidopsis thaliana Glucosinolate (GSL) Metabolism
Source: Life (Basel). 2022 Feb 22;12(3):326. doi: 10.3390/life12030326 (PMC8953324; doi:10.3390/life12030326)
Supplement: Supplementary file 1 [file life-12-00326-s001.zip › life-1594535-supplementary Figure S1.pdf]

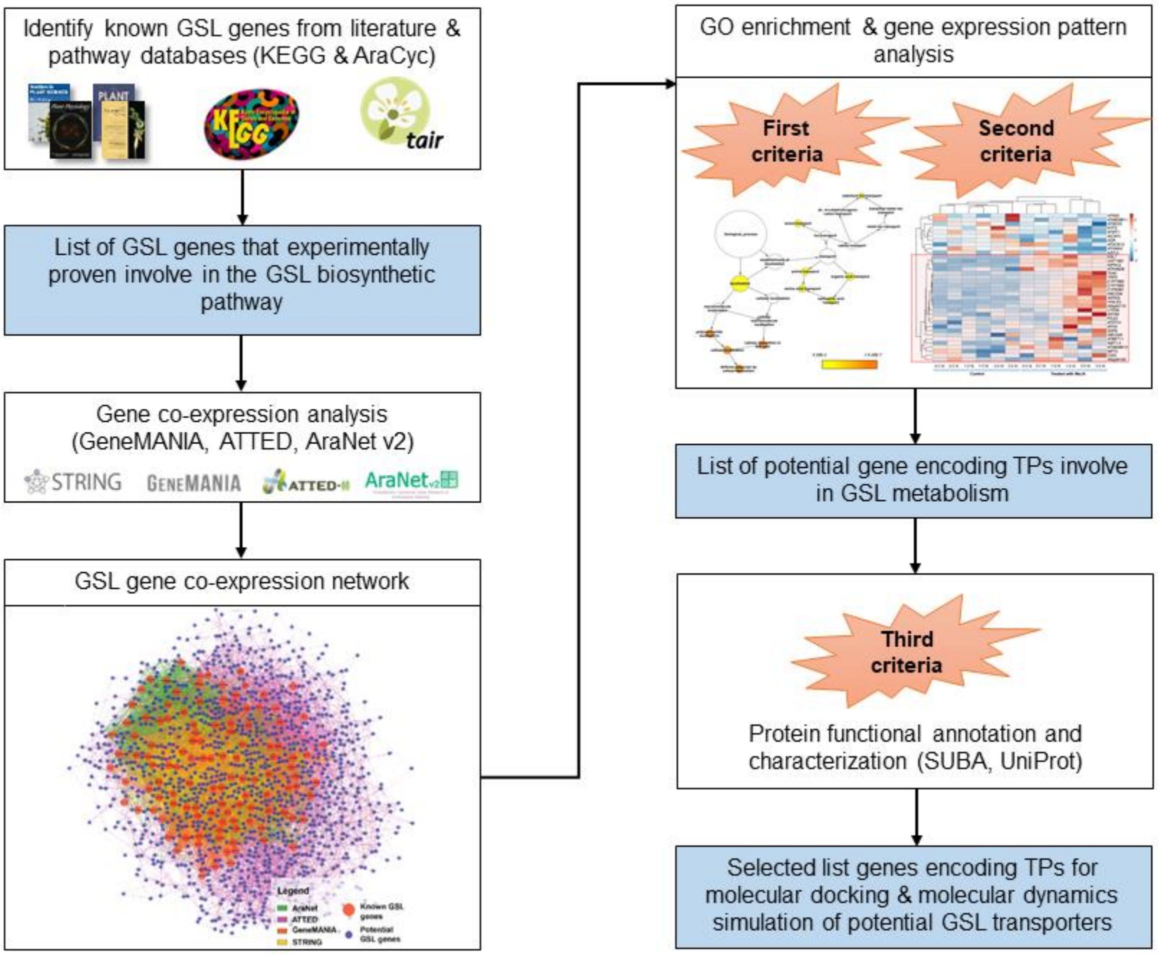

Supplementary Figure S1. The step-by-step procedure was designed in this study to identify potential genes encoding GSL TPs.
