# Supplementary material for: Identification of Potential Genes Encoding Protein Transporters in Arabidopsis thaliana Glucosinolate (GSL) Metabolism
Source: Life (Basel). 2022 Feb 22;12(3):326. doi: 10.3390/life12030326 (PMC8953324; doi:10.3390/life12030326)
Supplement: Supplementary file 1 [file life-12-00326-s001.zip › life-1594535-supplementary Table S1.pdf]

Supplementary Table S1. List of known GSL genes identified from the literature, AraCyc, and KEGG.

No.	TAIR_ID	Uniprot ID	Gene name	Protein name	Source		
					Literature	AraCyc	KEGG
1	AT1G16400	Q9FUY7	CYP79F2	Hexahomomethionine N-hydroxylase	√	√	√
2	AT1G16410	Q949U1	CYP79F1	Dihomomethionine N-hydroxylase	√	√	√
3	AT1G18590	Q9FZ80	SOT17	Cytosolic sulfotransferase 17	√	√	√
4	AT1G24100	O48676	UGT74B1	UDP-glycosyltransferase 74B1	√	√	√
5	AT1G74090	Q9C9C9	SOT18	Cytosolic sulfotransferase 18	√	√	√
6	AT1G74100	Q9C9D0	SOT16	Cytosolic sulfotransferase 16	√	√	√
7	AT2G20610	Q9SIV0	SUR1	S-alkyl-thiohydroximate lyase SUR1	√	√	√
8	AT2G22330	Q501D8	CYP79B3	Tryptophan N-monooxygenase 2	√	√	√
9	AT2G43100	Q9ZW84	IPMI2	3-isopropylmalate dehydratase small subunit 2	√	√	√
10	AT3G49680	Q9M401	BCAT3	Branched-chain-amino-acid aminotransferase 3, chloroplastic	√	√	√
11	AT3G58990	Q9LYT7	IPMI1	3-isopropylmalate dehydratase small subunit 3	√	√	√
12	AT4G13430	Q94AR8	IIL1	3-isopropylmalate dehydratase large subunit, chloroplastic	√	√	√
13	AT4G13770	P48421	CYP83A1	Cytochrome P450 83A1	√	√	√
14	AT4G31500	O65782	CYP83B1	Cytochrome P450 83B1	√	√	√
15	AT4G39950	O81346	CYP79B2	Tryptophan N-monooxygenase 1	√	√	√
16	AT5G05260	Q9FLC8	CYP79A2	Phenylalanine N-monooxygenase	√	√	√
17	AT5G14200	Q9FMT1	IMDH1	3-isopropylmalate dehydrogenase 1, chloroplastic	√	√	√
18	AT5G23010	Q9FG67	MAM1	Methylthioalkylmalate synthase 1, chloroplastic	√	√	√

19	AT5G23020	Q9FN52	MAM3	Methylthioalkylmalate synthase 3, chloroplastic	√	√	√
20	AT1G12140	A8MRX0	FMO GS-OX5	Flavin-containing monooxygenase FMO GS-OX5	√	√	
21	AT1G21100	Q9LPU5	IGMT1	Indole glucosinolate O-methyltransferase 1	√	√	
22	AT1G21120	Q9LPU7	IGMT2	Indole glucosinolate O-methyltransferase 2	√	√	
23	AT1G47600	Q8GRX1	BGLU34	Myrosinase 4	√	√	
24	AT1G51470	Q3ECS3	BGLU35	Myrosinase 5	√	√	
25	AT1G54040	Q8RY71	ESP	Epithiospecifier protein	√	√	
26	AT1G58260	Q9LQB7	CYP79C2	Cytochrome P450, putative	√	√	
27	AT1G62540	Q94K43	FMO GS-OX2	Flavin-containing monooxygenase FMO GS-OX2	√	√	
28	AT1G62560	Q9SXE1	FMO GS-OX3	Flavin-containing monooxygenase FMO GS-OX3	√	√	
29	AT1G62570	Q93Y23	FMO GS-OX4	Flavin-containing monooxygenase FMO GS-OX4	√	√	
30	AT1G65860	Q9SS04	FMO GS-OX1	Flavin-containing monooxygenase FMO GS-OX1	√	√	
31	AT1G76790	Q9SRD4	IGMT5	Indole glucosinolate O-methyltransferase 5	√	√	
32	AT1G79370	F4IF38	CYP79C1	Cytochrome P450, family 79, subfamily C, polypeptide 1	√	√	
33	AT2G25450	Q9SKK4	GSL-OH	Probable 2-oxoacid dependent dioxygenase	√	√	
34	AT2G33070	O49326	NSP2	Nitrile-specifier protein 2	√	√	
35	AT2G44490	O64883	PEN2	Beta-glucosidase 26, peroxisomal	√	√	
36	AT3G16390	O04318	NSP3	Nitrile-specifier protein 3	√	√	
37	AT3G16400	Q9SDM9	NSP1	Nitrile-specifier protein 1	√	√	
38	AT3G16410	O04316	NSP4	Nitrile-specifier protein 4	√	√	
39	AT3G44300	P32962	NIT2	Nitrilase 2	√	√	
40	AT3G44310	Q8LFU8	NIT1	Nitrilase 1	√	√	
41	AT3G44320	P46010	NIT3	Nitrilase 3	√	√	

42	AT4G03050	Q9ZTA1	AOP3	2-oxoglutarate-dependent dioxygenase AOP3	√	√	
43	AT4G03060	Q9ZTA2	AOP2	Probable inactive 2-oxoglutarate-dependent dioxygenase AOP2	√	√	
44	AT4G30530	Q9M0A7	GGP1	Gamma-glutamyl peptidase 1	√	√	
45	AT4G37400	Q0WTF4	CYP81F3	Cytochrome P450 81F3	√	√	
46	AT4G37410	Q9SZU1	CYP81F4	Cytochrome P450 81F4	√	√	
47	AT4G37430	O65790	CYP91A2	Cytochrome P450 81F1	√	√	
48	AT5G22300	P46011	NIT4	Bifunctional nitrilase/nitrile hydratase NIT4	√	√	
49	AT5G25980	Q9C5C2	TGG2	Myrosinase 2	√	√	
50	AT5G26000	P37702	TGG1	Myrosinase 1	√	√	
51	AT5G48180	Q93XW5	NSP5	Nitrile-specifier protein 5	√	√	
52	AT5G57220	Q9LVD6	CYP81F2	Cytochrome P450 81F2	√	√	
53	AT3G19710	Q9LE06	BCAT4	Methionine aminotransferase BCAT4	√		√
54	AT1G12130	Q9FWW3	AT1G12130	Flavin-containing monooxygenase FMO GS-OX-like 6	√		
55	AT1G12160	Q9FWW6	AT1G12160	Flavin-containing monooxygenase FMO GS-OX-like 1	√		
56	AT1G03400	Q94A78	AT1G03400	1-aminocyclopropane-1-carboxylate oxidase homolog 4		√	
57	AT1G03410	Q43383	2A6	1-aminocyclopropane-1-carboxylate oxidase homolog 5		√	
58	AT1G04350	P93824	AT1G04350	1-aminocyclopropane-1-carboxylate oxidase homolog 6		√	
59	AT1G04380	P93821	AT1G04380	1-aminocyclopropane-1-carboxylate oxidase homolog 7		√	
60	AT1G06620	Q84MB3	AT1G06620	1-aminocyclopropane-1-carboxylate oxidase homolog 1		√	

61	AT1G06640	Q9C5K7	AT1G06640	1-aminocyclopropane-1-carboxylate oxidase homolog 2		√		
62	AT1G06645	F4IDQ2	AT1G06645	2-oxoglutarate (2OG) and Fe (II)-dependent oxygenase superfamily protein		√		
63	AT1G06650	Q8H1S4	AT1G06650	1-aminocyclopropane-1-carboxylate oxidase homolog 3		√		
64	AT1G21110	Q9LPU6	IGMT3	Indole glucosinolate O-methyltransferase 3		√		
65	AT1G21130	Q9LPU8	IGMT4	Indole glucosinolate O-methyltransferase 4		√		
66	AT1G51490	Q9C8K1	BGLU36	Putative myrosinase 6		√		
67	AT1G58265	Q3E7Q8	AT1G58265	Cytochrome P450 superfamily protein		√		
68	AT1G62600	Q94BV5	AT1G62600	Flavin-containing monooxygenase FMO GS-OX-like 4		√		
69	AT1G66540	Q0WVW6	AT1G66540	Cytochrome P450 superfamily protein		√		
70	AT2G23190	O22188	CYP81D7	Cytochrome P450, family 81, subfamily D, polypeptide 7		√		
71	AT2G23220	O22185	CYP81D6	Cytochrome P450, family 81, subfamily D, polypeptide 6		√		
72	AT2G23960	F4INN2	GGP4	Gamma-glutamyl peptidase 4		√		
73	AT2G23970	O82225	GGP5	Gamma-glutamyl peptidase 5		√		
74	AT2G24850	Q9SK47	TAT3	Probable aminotransferase TAT3		√		
75	AT2G30830	O80850	AT2G30830	2-oxoglutarate		√		
76	AT2G30840	O80851	AT2G30840	2-oxoglutarate		√		
77	AT3G07720	Q9S7W4	F17A17.6	AT3g07720/F17A17_6		√		
78	AT3G61400	Q9M2C4	AT3G61400	1-aminocyclopropane-1-carboxylate oxidase homolog 8		√		
79	AT4G28410	F4JL94	RSA1	Tyrosine transaminase family protein		√		

80	AT4G28420	Q67Y55	AT4G28420	Probable aminotransferase TAT1		√	
81	AT4G30540	Q9M0A6	GGP2	Gamma-glutamyl peptidase 2		√	
82	AT4G30550	Q9M0A5	GGP3	Gamma-glutamyl peptidase 3		√	
83	AT4G37320	O23156	CYP81D5	Cytochrome P450, family 81, subfamily D, polypeptide 5		√	
84	AT5G07800	Q9FF12	AT5G07800	Flavin-containing monooxygenase FMO GS-OX-like 9		√	
85	AT5G35917	A0A178UFP3	CYP79A3P	CYP79A3P		√	
86	AT5G35920	Q9LTK9	CYP79A4P	Cytochrome P450-like		√	
87	AT5G36220	Q9FG65	CYP81D1	Cytochrome P450 81D1		√	
88	AT5G43440	Q9LSW7	AT5G43440	1-aminocyclopropane-1-carboxylate oxidase homolog 9		√	
89	AT5G43450	Q9LSW6	AT5G43450	1-aminocyclopropane-1-carboxylate oxidase homolog 10		√	
90	AT5G48375	Q3E8E5	TGG3	Putative myrosinase 3		√	
91	AT5G53810	Q9FHZ5	MGN6.20	Caffeic acid 3-O-methyltransferase-like protein		√	
92	AT5G59530	Q9LTH8	AT5G59530	1-aminocyclopropane-1-carboxylate oxidase homolog 11		√	
93	AT5G59540	Q9LTH7	AT5G59540	1-aminocyclopropane-1-carboxylate oxidase homolog 12		√	
94	AT5G67310	Q8GZ20	CYP81G1	At5g67310		√	
95	AT1G12200	Q9FWW9	FMO	Flavin-containing monooxygenase FMO GS-OX-like 2		√	
96	AT1G15540	Q9M9D9	AT1G15540	2-oxoglutarate-dependent dioxygenase-like protein		√	
97	AT1G28030	Q9C7F0	AT1G28030	2-oxoglutarate		√	
98	AT1G31180	Q9SA14	IMDH3	3-isopropylmalate dehydrogenase 3, chloroplastic		√	

99	AT1G52790	Q9C939	AT1G52790	2-oxoglutarate (2OG and Fe II)-dependent oxygenase superfamily protein		√	
100	AT1G52800	Q9C938	AT1G52800	2-oxoglutarate (2OG and Fe II)-dependent oxygenase superfamily protein		√	
101	AT1G52810	Q9C937	AT1G52810	2-oxoglutarate (2OG and Fe II)-dependent oxygenase superfamily protein		√	
102	AT1G52820	Q9C936	AT1G52820	2-oxoglutarate (2OG and Fe II)-dependent oxygenase superfamily protein		√	
103	AT1G62580	Q9SXD9	NOGC1	Flavin-containing monooxygenase FMO GS-OX-like 7		√	
104	AT1G62620	Q9SXD5	AT1G62620	Flavin-containing monooxygenase FMO GS-OX-like 3		√	
105	AT1G63340	Q9C8T8	AT1G63340	Putative flavin-containing monooxygenase FMO GS-OX-like 10		√	
106	AT1G63370	Q9C8U0	AT1G63370	Flavin-containing monooxygenase FMO GS-OX-like 5		√	
107	AT1G63390	Q9SH25	AT1G63390	Putative flavin-containing monooxygenase FMO GS-OX-like 11		√	
108	AT1G77530	Q9CAQ3	AT1G77530	O-methyltransferase family protein		√	
109	AT1G80320	Q9C972	AT1G80320	2-oxoglutarate		√	
110	AT1G80560	P93832	IMDH2	3-isopropylmalate dehydrogenase 2, chloroplastic		√	
111	AT4G03070	Q9ZTA3	AOP1	Probable 2-oxoglutarate-dependent dioxygenase AOP1		√	
112	AT5G37170	F4K5W7	MJG14.10	O-methyltransferase family protein		√	
113	AT5G61290	Q9FLK4	AT5G61290	Flavin-containing monooxygenase FMO GS-OX-like 8		√	
114	AT1G10060	Q93Y32	BCAT1	Branched-chain-amino-acid aminotransferase 1, mitochondrial			√

115	AT1G10070	Q9M439	BCAT2	Branched-chain-amino-acid aminotransferase 2, chloroplastic			√
116	AT5G65780	Q9FYA6	BCAT5	Branched-chain-amino-acid aminotransferase 5, chloroplastic			√
