# Supplementary material for: Identification of Potential Genes Encoding Protein Transporters in Arabidopsis thaliana Glucosinolate (GSL) Metabolism
Source: Life (Basel). 2022 Feb 22;12(3):326. doi: 10.3390/life12030326 (PMC8953324; doi:10.3390/life12030326)
Supplement: Supplementary file 1 [file life-12-00326-s001.zip › life-1594535-supplementary Table S2.pdf]

Supplementary Table S2. List of potential GSL transporters based on gene expression pattern with known GSL genes and the biological processes involved.

No.	Gene name	Synonym	UniProt ID	AGI ID	Type of gene	Protein localization	Protein name
1	ESL1 [LT]	SUGTL2	Q94KE0	At1g08920	Potential GSL transporter	Vacuole; a transporter for monosaccharides	Sugar transporter ESL1 (Protein EARLY-RESPONSIVE TO DEHYDRATION 6-LIKE 1)
2	AtPNC2 [LT]	PNC2	Q8VZS0	At5g27520	Potential GSL transporter	Peroxisome; involved in fatty acid beta-oxidation during early stage of postgerminative growth	Peroxisomal adenine nucleotide carrier 2
3	AtRAB2B [LT]	AtRABB1A	O23561	At4g17160	Potential GSL transporter	Mitochondrion peroxisome	Ras-related protein RABB1a
4	TAAC [LT]	At5g01500	Q9M024	At5g01500	Potential GSL transporter	Plastid; involved in providing ATP during thylakoid biogenesis and turnover	Thylakoid ATP/ADP carrier
5	TRP3 [L]	TSA1	Q42529	At3g54640	Potential GSL gene	Plastid; catalyzes the conversion of indole-3-glycerolphosphate to indole	Tryptophan synthase alpha chain
6	AERD2 [LT]	ERD2	P35402	At1g29330	Potential GSL transporter	Golgi; similar in sequence to animal and yeast endoplasmic reticulum retention signal receptor	ER lumen protein retaining receptor family protein
7	VHA-E3 [LT]	At1g64200	P0CAN7	At1g64200	Potential GSL transporter	Vacuole	Vacuolar H+-ATPase subunit E isoform 3
8	At5g02170 [LT]	AVT1E	Q8LPF4	At5g02170	Potential GSL transporter	Plasma membrane; transmembrane amino acid transporter family protein	Amino acid transporter AVT1E

9	LTPG6 [L]	At1g55260	F4I082	At1g55260	Potential GSL transporter	Cytosol	Bifunctional inhibitor/lipid-transfer protein/seed storage 2S albumin superfamily protein
10	AST68 [LT]	SULTR2;1	O04722	At5g10180	Potential GSL transporter	Plasma membrane	Sulfate transporter 2;1
11	PILS3 [LT]	At1g76520	Q9C9K5	At1g76520	Potential GSL transporter	Vacuole	Auxin efflux carrier family protein
12	AtDTX1 [LT]	DTX1	Q9SIA5	At2g04040	Potential GSL transporter	Plasma membrane; Detoxifying efflux carrier for plant-derived antibiotics and other toxic compounds, including CD2+	Protein DETOXIFICATION 1
13	ABCG40 [LT]	PDR12	Q9M9E1	At1g15520	Potential GSL transporter	Plasma membrane; involved in ABA transport and resistance to lead	ABC transporter G family member 40
14	AtBET11 [LT]	AtBS14A	Q9M2J9	At3g58170	Potential GSL transporter	Golgi; suppresses the temperature-sensitive growth defect in sft1-1 yeast cells	BET1P/SFT1P-like protein 14A
15	AMT1;4 [LT]	At4g28700	Q9SVT8	At4g28700	Potential GSL transporter	Plasma membrane	Ammonium transporter 1;4
16	AtMEMB12 [LT]	MEMB12	Q9FK28	At5g50440	Potential GSL transporter	Golgi	Membrin 12
17	MPT2 [LT]	PHT3;2	Q9M2Z8	At3g48850	Potential GSL transporter	Mitochondrion	Mitochondrial phosphate carrier protein 2
18	CDI3 [LT]	SLAC1	Q9LD83	At1g12480	Potential GSL transporter	Plasma membrane; expressed in guard cells that plays a role in regulating cellular ion homeostasis and S-type anion currents	Guard cell S-type anion channel SLAC1
19	At5g38160 [L]	MXA21.18	Q9FF40	At5g38160	Potential GSL transporter	Extracellular	Bifunctional inhibitor/lipid-transfer protein/seed storage 2S albumin superfamily protein

20	AFH3 [L]	FH3	F4JJE8	At4g15200	Potential GSL gene	Extracellular; actin nucleation factor that directs the formation of actin cables in pollen tubes	Formin 3
21	SDP6 [L]	At3g10370	Q9SS48	At3g10370	Potential GSL gene	Mitochondrion; possibly involved in storage lipid catabolism and glycerol assimilation	Glycerol-3-phosphate dehydrogenase SDP6

[L] genes that are involved in localisation, [T] genes that are involved in transport
